# Understanding female smoking in urban China: motivations, stigma and shifting social norms—a qualitative focus group study

**DOI:** 10.1136/bmjopen-2025-110684

**Published:** 2026-01-30

**Authors:** Xiaoyun Xie, Xinbo Di, Xi Yin, Wanjia He, Sophia Siu Chee Chan, Lin Xiao

**Affiliations:** 1 Tobacco Control Office, Chinese Center for Disease Control and Prevention, Beijing, China; 2 School of Nursing, The University of Hong Kong, Hong Kong, Hong Kong; 3 West Pacific Region, World Health Organization, Manila, Philippine; 4 School of Public Health, The University of Hong Kong, Hong Kong, Hong Kong

**Keywords:** Tobacco Use, Social Cognition, China

## Abstract

**Abstract:**

**Background:**

Although female smoking prevalence in China remains low, emerging evidence suggests that social acceptance may be increasing, with tobacco marketing increasingly targeting women. This study explored women’s smoking behaviours, motivations and societal perceptions toward this in urban China.

**Methods:**

Between May and October 2019, 28 semistructured focus groups were conducted in Beijing, Changsha and Shenzhen with 288 participants: 12 groups of female smokers, 6 of female former smokers, 6 of female never-smokers and 4 of men. Participants were recruited both online and offline, and smoking status was verified with a carbon monoxide monitor. Discussions were transcribed verbatim and analysed thematically using dual coding.

**Results:**

Four themes emerged. First, while stigma against female smoking persisted, social acceptance is growing, especially among younger generation. Second, three initiation stages were identified: adolescence, early career and post-retirement, often triggered by peer influence, occupational stress and life transitions. Third, many female smokers concealed their behaviour, reflecting tension between shifting descriptive norms and enduring injunctive norms, which may contribute to underreporting in surveillance data. Fourth, misconceptions about smoking harms and quitting were common, with most women who smoke relying on willpower and showing limited interest in cessation support; pregnancy was one of the few strong motivators for quitting.

**Conclusions:**

Findings suggest gradual normalisation of female smoking in urban China, driven by evolving gender roles and targeted marketing. Public health responses should prioritise gender-specific health education, strengthen promotion of cessation services and tighten restrictions on tobacco marketing towards women to prevent future increases in female smoking.

**STRENGTHS AND LIMITATIONS OF THIS STUDY**

- This qualitative study included a large and diverse sample (n=288 across three major cities).
- Data collection and analysis were conducted by trained researchers following a predefined analytic framework to enhance methodological rigour.
- As the study was conducted in urban settings, the findings may not be transferable to rural areas with different social norms and tobacco environments.
- The data were collected in 2019, and changes in societal norms or tobacco industry strategies since then may limit the contemporary relevance of the findings.

## Introduction

 Smoking remains the leading preventable cause of death globally, responsible for around 7 million deaths annually, including approximately 1.51 million women.^1^ Beyond the well-recognised risks of cancer, cardiovascular disease and chronic respiratory illness,^2^ women who smoke face sex-specific harms—including cervical cancer, osteoporosis, infertility and earlier menopause^3^—and are more susceptible than men to lung cancer and Chronic Obstructive Pulmonary Disease (COPD) at equivalent exposure levels.^4^ Maternal smoking further heightens the risk of miscarriage, pre-term delivery, low birth weight and neonatal morbidity.^5^ These distinct vulnerabilities underscore the importance of gender-responsive tobacco-control strategies.

Over the past half-century, the traditional male predominance in smoking has narrowed in many high-income countries (HICs) through a predictable diffusion pattern: men adopt first and peak early, while women’s uptake accelerates later, often catalysed by industry marketing that links smoking with emancipation and modern femininity.^6 7^ In several HICs—including the UK—female smoking plateaued only recently and still rises within some sub-groups despite overall declines.^8^ Cross-national analyses show that higher gross national income and greater gender empowerment are each associated with higher female-to-male smoking prevalence ratios, suggesting that economic and social advances can facilitate smoking among women unless counter-balanced by effective policy.^9^ Similar trajectories are now anticipated in many low- and middle-income countries (LMICs) as women’s spending power and social autonomy expand.^10^

The tobacco industry has responded by aggressively targeting women in LMICs.^11^,^13^ In East and South-East Asia, marketing campaigns promote slim, flavoured and ‘light’ cigarettes in elegant packaging, framing smoking as fashionable, independent and sophisticated.^14^ China represents a vast untapped market with around 650 million women. Although male smoking prevalence remains among the world’s highest (43.9 %), only 1.8% of adult women currently smoke, yielding one of the globe’s widest sex gaps.^15^ This disparity is widely attributed to entrenched cultural norms that stigmatise female smoking.^16^ Yet the rapid rise in female smoking seen in culturally similar neighbours such as Japan^17^ and the Republic of Korea^18^ illustrates how these norms can erode when economic development, westernisation and targeted marketing converge. Signs of such erosion are emerging in China: female smoking has become more acceptable in China^19^; the smoking rate among middle school girls has increased in most provinces in China^20^; consecutive nationally representative studies also identified an upward trend in smoking prevalence among women in China who were born after the 1990s and those with higher education^21^—figures that are climbing alongside urbanisation and pervasive digital advertising.

Given the potentially substantial public health impact of increased female smoking in China, it is crucial to monitor smoking trends and to understand societal perceptions and norms regarding female tobacco use. Routine surveillance quantifies prevalence but offers little insight into how Chinese women interpret smoking within shifting gender roles, or how descriptive (‘what people do’) and injunctive (‘what people approve’) norms shape behaviour. Qualitative evidence on women’s lived experiences, motivations and responses to gender-specific marketing is sparse. Understanding these sociocultural determinants is critical for preserving China’s exceptionally low female prevalence and forestalling a foreseeable rise.

This qualitative study aims to explore smoking behaviours and motivations among Chinese women and to examine adult smokers’ and non-smokers’ perceptions of female tobacco use. Understanding these sociocultural determinants is critical for anticipating future changes in prevalence and developing gender-responsive tobacco control strategies in China.

## Methods

### Study design and participants

Semi-structured focus groups were conducted to explore women’s tobacco use behaviours, beliefs, attitudes, perceptions and motivations for both using and quitting tobacco, as well as both men’s and women’s perceptions of female tobacco use. Four participant categories were defined based on the following inclusion criteria:

1. Female smokers: Adult women (≥18 years) who currently use any tobacco product (smoking or smokeless), either daily or non-daily.
2. Female never-smokers: Adult women (≥18 years) who have never experimented with any tobacco products.
3. Female ex-smokers: Adult women (≥18 years) who previously used any tobacco product (smoking or smokeless) but were not using any at the time of recruitment.
4. Men: Adult men (≥18 years), irrespective of their smoking status.

Across the four interview categories, we purposively formed multiple groups, with half of the groups comprising participants aged 18–30 and the remaining half comprising participants over 30. To ensure relevance and depth, each participant category was interviewed using a slightly tailored interview guide. The interview guide was developed based on our previous qualitative research with female smokers in Beijing and refined through consultation with experts from the US Centres for Disease Control and Prevention (CDC). The guide was then pilot tested with several women who smoke, women who do not smoke and men in Beijing to assess clarity, relevance and flow. Feedback from the pilot interviews informed further revisions. The key topics covered for each category are summarised in table 1, and the full interview guides have been provided as onlinesupplemental files 1^4^.

**Table 1:** Participant categories and corresponding focus group topics

Focus group	Topics covered
Women (smokers)	Initiation, motivations to use, cessation attempts (facilitators, barriers), perceptions of female tobacco users
Women (never-smokers)	Motivations to avoid, considerations of initiation (facilitators, barriers), perceptions of female tobacco users
Women (ex-smokers)	Initiation, former motivations to use, cessation attempts (facilitators, barriers), successful quit attempts, perceptions of female tobacco users
Men	Perceptions of female tobacco users, perceptions of tobacco use

### Procedure

Data collection took place in Beijing, Changsha and Shenzhen and was facilitated by the Chinese Center for Disease Control and Prevention (CCDC). Recruitment of participants was conducted through WeChat, a widely used mobile messaging platform in China, in combination with referrals from local community organisations to ensure broader outreach and diversity of participants. Each focus group was facilitated by an experienced female qualitative expert (JZ), with a female note-taker (XBD) and recorder present.

### Data analysis

The focus group transcripts and accompanying field notes were analysed using thematic analysis, as described by Braun and Clarke.^22^ First, two researchers (XX and XD) familiarised themselves with the data by reading all the transcripts line-by-line to generate initial thoughts on the data. A deductive coding framework was developed based on the focus group guides and applied to the transcripts. During coding, the analysts inductively generated additional descriptive codes to capture emergent topics. The coding framework was iteratively refined through team discussions, and overlapping or conceptually similar codes were revised, merged or redefined as needed. Broader themes were then identified and defined by collating conceptually similar subcategories across the four participant groups and across all focus groups.

All transcripts were managed and coded in NVivo, and theme development was led by the two analysts in consultation with the wider research team. The analysis team consisted of both male and female public health researchers trained in qualitative methods and tobacco control. To enhance reflexivity, the team discussed potential biases throughout the analytic process and grounded theme development closely in participants’ accounts. Analyses were performed in the original Chinese; selected interview excerpts were translated into English for reporting. We followed the Standards for Reporting Qualitative Research (SRQR) reporting guideline.^23^

## Results

### Sample characteristics

A total of 288 participants were interviewed in 28 focus groups (8 to 13 participants each) across three cities from May to October 2019. Each interview lasted about 2 hours. Table 2 shows the details of the focus groups. City information was omitted from quotation to protect participants’ privacy.

**Table 2:** Distribution of focus groups by city and participant composition

City	Women (smokers)	Women (never-smokers)	Women (ex-smokers)	Men	Total
Beijing	4	2	2	2	10
Changsha	4	2	2	2	10
Shenzhen	4	2	2	0	8
Total	12	6	6	4	28

### Attitudes toward female smoking

Our study found that although most participants believed that the prevalence of female smoking has increased and that social acceptance of female smoking is higher than it was decades ago, attitudes toward female smoking remain complex.

But it seems that smoking among women, especially younger women, has increased. At least in my mother’s generation, women didn’t smoke. But now I do see some women in their 30s or 40s who smoke, and it even comes across as quite ‘trendy’. (male, 30+)

People are more open now. I think young people don’t really care if women smoke. But the older generation, like my father’s generation, still hold the view that it’s acceptable for men to smoke but not for women. (female smoker, 30+)

Female smokers generally perceive smoking as a personal choice, asserting that women should have the same right to smoke as men, free from judgement.

People used to think smoking was a privilege for men. But I don’t see why women can’t smoke as well. (female smoker, 30+)

I think as long as it doesn’t affect others in public spaces, it’s totally fine for women to smoke. (female smoker, 18-30)

However, many female smokers reported experiencing negative comments or disapproving looks from strangers, particularly from older individuals.

I used to have a senior leader who thought it was fine for men to smoke but was very critical of women who smoked. A female colleague was even fired for smoking at work, while the men faced no consequences. (female smoker, 30+)

There was a man in his 50s who once said, ‘How dare girls smoke nowadays!’ and I ended up arguing with him. (female smoker, 18-30)

Some female never-smokers expressed negative views towards female smoking, sometimes rooted in stereotypes.

If I meet someone new and my first impression of her is really good, but then I find out she smokes, my impression of her would drop immediately. (female never-smoker,18-30)

Despite this, a significant number of female never-smokers found female smoking acceptable.

Smoking can look elegant and attractive, especially for women in senior positions. (female never-smoker, 30+)

If she’s older or a successful, mature woman under a lot of pressure, I can understand if she smokes or drinks sometimes. It may just be her way of coping with stress. (female never-smoker, 18-30)

My views have changed. I used to think girls who smoked weren’t good to be friends with. But after working and getting to know them, I found many female smokers are gentle and very capable. Smoking doesn’t define their personality or ability. (female never-smoker, 18-30)

Among male participants, attitudes were varied. While most men expressed acceptance of female smoking, some reversed their stance when it came to their girlfriend, wife or daughter smoking.

I don’t have any special opinions about women smoking. If men can smoke, then women can too. (male smoker, 30+)

I can’t accept my wife smoking. Whatever other women do is none of my business, but I just can’t accept it if it’s my wife. (male never-smoker, 18-30)

Generational differences in attitudes were particularly noticeable in mothers’ responses to their daughters’ smoking. Most female smokers reported that their parents, especially mothers, would likely oppose their smoking if they found out. However, many female smokers indicated that they would find it acceptable if their daughter were older than 18 years, and a number of female never-smokers agreed with this view.

My mom and dad are both smokers, but my mom told me that I should never smoke. (female smoker, 18-30)

Smoking is definitely unhealthy, but if she (my daughter) is an adult and chooses to smoke, I wouldn’t intervene. (female never-smoker, 18-30)

### Reasons why women start smoking

When discussing the reasons for smoking initiation among female smokers, three distinct life stages emerged: adolescence, early career and post-retirement.

- Rebellious teen: Participants who initiated smoking during adolescence were often influenced by smoking peers, viewing smoking as a means to build social connections. For some, smoking symbolises fashion, independence and courage. Attractive, gender-targeted cigarette packaging also contributed to initiation in this group.

When I was in junior high, several of my close friends smoked. One day in the bathroom they offered me a cigarette, and I felt too awkward to turn it down — that’s how I started smoking. (female smoker, 30+)

The cigarette packages were so beautiful that I started collecting them, and eventually, I ended up smoking too. (female smoker, 30+)

- Stressed professional: Many participants reported initiating smoking after entering the workforce, primarily as a coping strategy for work-related stress. Social influences from colleagues or friends who smoke also played a role.

I was really stressed after work, and since I couldn’t just drink to relax, I ended up smoking with my colleagues in the smoking corner. (female smoker, 18-30)

Everyone at work smoked, and if you didn’t join them, you’d seem like the odd one out. (female smoker, 30+)

- Empty-nest starter: A smaller group of women began smoking after retirement, often describing feelings of loneliness and life transitions following children becoming independent. Smoking was adopted as a way to cope with this perceived emptiness.

I started smoking after I retired. I picked it up gradually when I was hanging out with my friends. (female smoker, 30+)

Some older women smoke while chatting, and my mother-in-law told me that many of them only started smoking later in life. (female never-smoker, 30+, Beijing)

### Characteristics of female smoking

Our findings indicated that female smokers often concealed their smoking behaviour. Most reported that they did not want their parents to know they smoked and refrained from smoking in front of them, even if their parents were aware.

When I smoked at home, I had to make sure the ash and cigarette butts were cleaned up so my parents wouldn’t notice. (female smoker, 18-30)

I think my dad probably knows I smoke, but we’ve never talked about it. But I absolutely can’t let my mom find out — she would freak out. (female smoker, 30+)

Although most female smokers felt that their boyfriend or husband should accept their smoking, some participants still chose to hide it from their partners.

If I’m going to start a relationship, I’ll tell him that I smoke. He should accept who I am, If he can’t, then we just say goodbye*.* (female smoker, 30+)

Actually, even now, my husband still doesn’t know that I smoke. (female smoker, 30+)

Additionally, female smokers sometimes concealed their smoking behaviour around new acquaintances or non-smokers. However, they were more open about their smoking once trust had been established, and no strong disapproval was expressed.

Only two of my friends know that I smoke — no one else does. (female smoker, 30+)

I didn’t smoke in front of my friends at first. I only told them once we became close enough. (female smoker, 18-30)

Many female smokers enjoyed smoking with other smokers, especially friends and colleagues, as smoking served as a social connector and a way to relieve stress. However, some also tend to smoke alone, typically when surrounded by non-smokers or when trying to keep their smoking private. Many reported feeling uncomfortable smoking in front of children or pregnant women due to concerns about the harmful effects of passive smoking.

I don’t really smoke when I’m by myself — mainly when I’m with friends or out having fun. (female smoker, 18-30)

My friends don’t smoke, so I don’t smoke when I’m with them. I usually smoke on my own. (female smoker, 30+)

I have a personal rule: I don’t smoke around children, the elderly, or pregnant women. I don’t want my smoking to affect them. (female smoker, 18-30)

### Knowledge about the harms of smoking and attitudes toward quitting smoking

Most female smokers acknowledged the harmful effects of smoking on health, particularly on the lungs and fetus. However, some smokers minimised the risks, citing personal experiences or those of acquaintances as proof that smoking does not always lead to harm.

Many women with lung cancer never smoke, smoking may have something to do with lung cancer, but it’s not crucial. (female smoker, 30+)

From my own experience, smoking hasn’t affected my child’s well-being. My son is healthy and smart. (female smoker, 30+)

We also found that most female smokers were not particularly interested in quitting. Many young female smokers felt they had not yet experienced significant harm from smoking or believed they were too resilient to worry about health risks.

I know smoking is harmful, but it never really feels like it will happen to me. (female smoker, 18-30)

People keep talking about how harmful smoking is, but honestly, who cares? I just want to enjoy the moment. (female smoker, 18-30)

Among those who recognised the importance of quitting, many were deterred by perceived challenges, such as the fear of weight gain. Some believed that quitting after years of smoking could be detrimental to their health, as their bodies had adapted to smoking, and sudden cessation might cause harm.

I tried to quit this year, but gained a lot of weight afterwards, so I started smoking again to control my weight. (female smoker, 18-30)

I’ve heard more than once that when long-term heavy smokers quit abruptly, their health seems to decline, and they feel better only after smoking again. For people who’ve smoked for decades, their bodies feel dependent on it. (female smoker, 18-30)

Moreover, female smokers tended to believe that they could quit smoking easily with willpower alone. Most were reluctant to seek professional cessation services, as they did not view smoking as a disease and felt it was unusual to see a doctor for smoking-related issues. Most participants who had quit smoking reported not having sought professional help or using formal cessation methods.

All those quitting techniques are just nonsense. In the end, it’s really about your own determination. (female smoker, 18-30)

I don’t see the point of going to a clinic to get medication — it doesn’t really help. When I quit, I just kept myself busy, worked more, and once I had things to focus on, I didn’t even think about smoking. Plus, those meds can have side effects. (female ex-smoker, 18-30)

Pregnancy emerged as a strong motivator for quitting smoking. Nearly all female smokers stated they would seriously consider quitting when planning for or during pregnancy. Several participants who had quit confirmed that pregnancy was the key factor that led them to quit.

I’ve often wondered what it would take for me to quit, and honestly the only thing I can think of is getting pregnant. If I were pregnant, I’d definitely stop smoking, then I could quit naturally. (18-30 female smoker)

I quit when I was pregnant, but five or six years later I slowly started smoking again. My child was older by then, and I guess the cravings just came back. (female smoker, 30+)

## Discussion

Our study highlights a shift in societal attitudes toward female smoking in China, reflecting the weakening of traditional social and cultural constraints. Conventionally, female smoking in China has been stigmatised due to injunctive social norms that discouraged women from smoking, along with women’s limited economic resources.^19^ However, our findings suggest that, particularly in large cities such as Beijing, Changsha and Shenzhen, descriptive norms around female smoking are changing as with greater social acceptance. Younger women, in particular, were more open to the idea of future generations of women smoking compared with older generations, indicating a shift in societal attitudes. This generational difference may be linked to the profound changes in women’s roles in society, where they now play a larger part in the economic sphere. As younger generations reject traditional gender roles and embrace women’s emancipation, both injunctive and descriptive norms around female smoking seem to be transforming.

The increased acceptance of female smoking may also be partly attributed to the growing tobacco marketing targeting women.^13^ Advertising that associates smoking with women’s liberation, independence and equality with men has likely played a significant role in reshaping societal views.^11^ Some female smokers in our study reported smoking ‘feminised cigarette products’ and associated smoking with symbols of independence, which could be a direct result of such marketing campaigns. This suggests that social marketing of tobacco, which portrays smoking as a symbol of empowerment for women, may contribute to the normalisation of female smoking by altering both descriptive norms (by increasing visibility of female smokers) and injunctive norms (by reducing social disapproval).^9^

### Health implications and misconceptions

Our study found that most female smokers lacked sufficient motivation to quit, which may be due to a combination of misconceptions and lack of awareness about the risks of smoking. For example, some female smokers believed that quitting after years of smoking might be detrimental to their health, as their bodies had become accustomed to smoking, and sudden cessation could cause harm. These misconceptions highlight the need for improved health education and public awareness campaigns to address the specific health risks of smoking for women. Furthermore, it is worth noting that many female smokers in our study were motivated to quit smoking during pregnancy or due to concerns about the health effects of smoking on their children. This suggests that targeted campaigns emphasising the risks of smoking during pregnancy and its impact on offspring could be effective in motivating women to quit.

### Challenges in smoking cessation

The high addiction potential of nicotine presents a major barrier to quitting. Studies show that only 3–5% of smokers who attempt to quit on their own without professional help succeed in maintaining long-term abstinence.^24^ Moreover, research has shown that women may develop nicotine dependence more quickly than men and face greater challenges when trying to quit.^25 26^ Although the Chinese government has supported over 2000 cessation clinics, the 2018 Global Adult Tobacco Survey (GATS) China survey revealed that only 4% of female smokers have sought professional cessation services, including quitlines and cessation clinics.^27^ Our study indicates that female smokers’ attitudes towards cessation services may partly explain this low utilisation rate. Many female smokers in our study believed that quitting smoking depended solely on their willpower, and some viewed cessation techniques as ineffective. Additionally, a common belief was that it was unnecessary or even strange to seek medical help for quitting smoking. These beliefs further underline the need for public health campaigns aimed at changing attitudes towards smoking cessation services. Encouraging female smokers to view cessation as a health priority and not simply a matter of willpower is essential for increasing the use of cessation resources.

### Social and cultural barriers to smoking disclosure

Although societal attitudes toward female smoking have become more tolerant, many female smokers continue to conceal their smoking behaviour. This behaviour illustrates the tension between changing descriptive norms (more women are smoking) and persistent injunctive norms (female smoking is still viewed negatively by some). In our study, female smokers reported that they preferred not to disclose their smoking habits to their parents and avoided smoking in front of new acquaintances or non-smoking colleagues. These findings suggest that while the social stigma surrounding female smoking has lessened, it has not disappeared entirely. The persistence of these injunctive norms creates a situation where women must navigate conflicting social expectations. Many women still feel the need to protect themselves from negative judgments and criticism by concealing their smoking behaviour. This aligns with studies from South Korea, which found that self-reported data on female smoking was often underestimated, as women were reluctant to disclose their smoking status to family or neighbours.^28^ Given this, we recommend that future national tobacco surveys in China incorporate biochemical validation to obtain more accurate estimates of the female smoking rate.

### Smoking Initiation: timing and motivations

Our study also revealed that women initiate smoking at different stages in life and for various reasons, which can be understood through the lens of Social Norms theories. Smoking initiation during adolescence was typically influenced by peer pressure and the desire to fit in, with some participants viewing smoking as a symbol of fashion, independence and courage. From a social norms perspective, these young women are responding to perceived descriptive norms within their peer groups.^29^

For those who began smoking after entering the workforce, stress relief and socialising with colleagues were significant motivating factors.^30^ This reflects how workplace environments can create new descriptive norms that facilitate smoking adoption. In workplaces where smoking is common among colleagues, women may perceive smoking as normative behaviour for professional integration. Similarly, smoking initiation after retirement was often linked to feelings of emptiness, as women were freed from the responsibilities of raising children and work. These findings suggest that smoking prevention interventions should be tailored to address the specific needs of each group and the particular social norms operating in different life contexts. For example, targeting young women with messages about the long-term health risks of smoking and the availability of cessation services could help prevent smoking initiation by countering the perceived benefits of adoption. Peer interventions may also be effective, particularly in groups where women are more likely to smoke socially, as they can help reshape descriptive norms within these social networks.

### Implications for public health and tobacco control

Our study suggests that female smoking in China may be increasing subtly, with more women smoking than is often reported in national surveys. This potential under-reported increase in female smoking, combined with low levels of cessation service utilisation, presents significant public health challenges. To effectively address this issue, public health interventions should target both descriptive norms (by highlighting that most Chinese women do not smoke) and injunctive norms (by reinforcing positive social attitudes toward non-smoking women). With more than 2 million tobacco-related deaths in China each year, the rising rates of female smoking could have a profound impact on public health, particularly as China aims to achieve the goal of a Healthy China by 2030.

In light of our findings, we strongly advocate for targeted, gender-sensitive interventions to prevent smoking initiation among women and to improve female smokers’ intention of smoking cessation. These should include improved public health campaigns that leverage social norms approaches, better access to cessation services and efforts to address social and cultural barriers that continue to limit women’s willingness to seek help. Communication strategies should be designed to address the specific innovation attributes that make smoking attractive to different segments of women. Ultimately, reducing female smoking is a critical component of China’s broader efforts to improve public health and achieve its Healthy China objectives.

### Limitations

This study was conducted in three major Chinese cities—Beijing, Changsha and Shenzhen. Therefore, the findings are primarily applicable to urban contexts and may not be generalisable to smaller cities or rural areas, where patterns of female smoking could differ. Besides, the data were collected in 2019, prior to the COVID-19 pandemic. Social norms, patterns of tobacco use and public attitudes toward women’s smoking may have evolved in the intervening years, which limits the contemporaneous relevance of some findings. The delay in analysis and manuscript preparation was partly due to the scope of the qualitative dataset, competing research commitments and disruptions associated with the pandemic. Additionally, our study did not collect sufficient information on other tobacco products, such as electronic cigarettes or heated tobacco products, which limits our understanding of the complete landscape of tobacco use among Chinese women.

## Conclusions

Female smoking in urban China is becoming more socially acceptable, particularly among younger women, yet stigma persists and many continue to conceal their behaviour. Initiation often occurs at adolescence, early career or post-retirement, with stress, peer influence and life transitions as key drivers. Misconceptions about health risks and reliance on willpower contribute to low use of cessation services. Gender-sensitive interventions that counter industry marketing, correct misconceptions and improve access to cessation support are essential to prevent a rise in female smoking.

## Supplementary material

10.1136/bmjopen-2025-110684 online supplemental file 1

10.1136/bmjopen-2025-110684 online supplemental file 2

10.1136/bmjopen-2025-110684 online supplemental file 3

10.1136/bmjopen-2025-110684 online supplemental file 4

## Data Availability

Data are available upon reasonable request.
