# Supplementary material for: Understanding female smoking in urban China: motivations, stigma and shifting social norms—a qualitative focus group study
Source: BMJ Open. 2026 Jan 30;16(1):e110684. doi: 10.1136/bmjopen-2025-110684 (PMC12863354; doi:10.1136/bmjopen-2025-110684)
Supplement: online supplemental file 2 [file bmjopen-16-1-s002.docx]

**Female Never-Smoker Focus Group Interview Guide**

**(90–120 minutes)**

**Opening Script**

We’ve already gone over the ground rules, so let’s begin the session.
To help us get comfortable, I’d like to go around the room and have each of you share your surname and your favorite food.

I’ll start. My name is ________, and my favorite food is ________.

(Moderator moves around the room to ease the atmosphere.)

······

(Moderator resumes)

Thank you for the interesting answers! You’ve made me curious to try some of the dishes you mentioned.

*If this has not been covered naturally in the introductions, probe as needed.*

Now, let’s begin our discussion.

**Section I. General Health**

1. In general, how would you describe your overall health?
2. What health issues do you currently experience?
3. When you have a health concern, whom do you usually turn to for advice or support?
   *Probe: How do they help you?*
4. Thinking about people around you, how would you describe their health? What kinds of health problems do you think they face?
5. What do you think is the relationship between smoking and health?

**Section II. Smoking**

1. Tell me your views on smoking.
2. What are the smoking experiences of the people around you?
   *Probe: Family members, friends, colleagues.*
   A. How do you feel about their smoking?

**Section III. Women and Smoking**

1. Tell me your views on women and smoking.
2. What do you think of women who smoke?
3. In your opinion, what makes women’s smoking unacceptable?
4. How do people around you view women who smoke?
   *Probe: Family, friends; acceptance or disapproval.*
5. In China, how has women’s smoking behavior changed over time?
   A. In what ways has it changed?
   *Probe: Visibility in public spaces, social norms, etc.*

**Section IV. Smoking Behavior (Non-Smoker Perspective)**

1. What were the reasons you chose not to start smoking?
2. What factors influenced your decision not to smoke?
3. How have other people affected or shaped your decision not to smoke?
4. In what situations have people tried to persuade or encourage you to smoke?

**Section V. Quitting Smoking**

1. When do you think women who smoke should stop smoking?
   *Probe: Pregnancy, getting older, health concerns, financial considerations, marriage.*
2. What do you think are the most effective ways to encourage women to quit smoking?
3. What are the best ways to prevent women from starting to smoke in the first place?

**Section VI. Final Questions**

1. Tell me what you know about women’s use of smokeless tobacco.
2. What do you know about e-cigarettes?
