# Supplementary material for: Understanding female smoking in urban China: motivations, stigma and shifting social norms—a qualitative focus group study
Source: BMJ Open. 2026 Jan 30;16(1):e110684. doi: 10.1136/bmjopen-2025-110684 (PMC12863354; doi:10.1136/bmjopen-2025-110684)
Supplement: online supplemental file 3 [file bmjopen-16-1-s003.docx]

**Female Smoker Focus Group Interview Guide**

**(90–120 minutes)**

**Opening Script**

We’ve already gone over the ground rules, so let’s begin the session.
To help us get comfortable, I’d like to go around the room and have each of you share your surname and your favorite food.

I’ll start. My name is ________, and my favorite food is ________.

(Moderator moves around the room to ease the atmosphere.)

······

(Moderator resumes)

Thank you for the interesting answers! You’ve made me curious to try some of the dishes you mentioned.

*If this has not been covered naturally in the introductions, probe as needed.*

Now, let’s begin our discussion.

**Section I. General Health**

1. In general, how would you describe your overall health?
2. What health issues do you currently experience?
3. When you have a health concern, whom do you usually turn to for advice or support?
   *Probe: How do they help you?*
4. Thinking about people around you, how would you describe their health? What kinds of health problems do you think they face?
5. What do you think is the relationship between smoking and health?

**Section II. Smoking**

1. What do you think are the benefits of smoking, if any?
2. What do you think are the harms of smoking?
3. What are the smoking experiences of people around you?
   *Probe: Men, women, family members, friends, colleagues.*
   A. How do you feel about their smoking?
4. In China, how has women’s smoking behavior changed over time?
   A. In what ways has it changed?
   *Probe: Visibility in public, changing social norms.*

**Section III. Initiating Smoking**

Thinking back to when you first started smoking:

1. What were the reasons you began smoking?
2. What influenced you to start smoking?
   *Probe: Was anything significant happening in your life at that time?*
3. Who were the people who influenced you to start smoking?
   *Probe: Family members who smoked, friends who smoked.*
4. Tell us about your first smoking experience—what happened?

**Section IV. Smoking Behavior**

1. What do you enjoy most about smoking?
   *Probe: What keeps you smoking now?*
2. What do you dislike most about smoking?
3. When you smoke, do you usually smoke alone or with others?
4. What challenges or difficulties do you face when smoking?
   *Probe: Finding places to smoke, hiding cigarettes, attitudes of family, friends, colleagues.*
5. What have others said to you about your smoking?
   *Probe: Family, friends, partner, colleagues.*
6. Around whom do you feel comfortable smoking?
   *Probe: Family, friends, partner, colleagues.*
7. Around whom do you feel uncomfortable smoking?
   *Probe: Family, friends, partner, colleagues.*

**Section V. Quitting Smoking**

1. What are the important reasons you would want to quit smoking?
2. Have you ever tried to quit smoking?
3. If you were to try quitting:
   – What do you think would be most helpful?
   – What would be least helpful?
   – What kind of support do you think you would need?
4. What do you think are the most effective ways to encourage women to quit smoking?
5. What do you think are the best ways to prevent women from starting to smoke?

**Section VI. Final Questions**

1. Tell me what you know about women’s use of smokeless tobacco.
2. What do you know about e-cigarettes?
