# Supplementary material for: Understanding female smoking in urban China: motivations, stigma and shifting social norms—a qualitative focus group study
Source: BMJ Open. 2026 Jan 30;16(1):e110684. doi: 10.1136/bmjopen-2025-110684 (PMC12863354; doi:10.1136/bmjopen-2025-110684)
Supplement: Supplementary file 5 [file bmjopen-2025-110684.reviewer_comments.pdf]

PEER REVIEW HISTORY

BMJ Open publishes all reviews undertaken for accepted manuscripts. Reviewers are asked to complete a checklist review form and are provided with free text boxes to elaborate on their assessment. These free text comments are reproduced below.

ARTICLE DETAILS

Title (Provisional)

Understanding female smoking in urban China: motivations, stigma, and shifting social norms-a qualitative focus group study

Authors

Xie, Xiaoyun; Di, Xinbo; Yin, Xi; He, Wanjia; Chan, Sophia Siu Chee; Xiao, Lin

---

VERSION 1 - REVIEW

**Reviewer**	**1**
**Name**	**He, Di**
**Affiliation**	**University of California Los Angeles Fielding School of Public Health**
**Date**	**20-Oct-2025**
**COI**	**None**

This manuscript presents a qualitative focus group study examining smoking behaviors, motivations, and societal perceptions among women in urban China. The authors conducted 28 semi-structured focus groups in 2019. The findings suggest a gradual normalization of female smoking in urban settings and discuss the public health implications, along with potential responses to promote health in this population and strengthen tobacco control efforts.

Overall, this is a very well-written and informative manuscript. I have the following minor comments.

Abstract

Please spell out any abbreviations when they first appeared, i.e. what does CO stand for in Page 1, line 12?

Introduction

Page 6, line 16: The smoking rates or smoking attempt rate were reported in publications in the 2010s, and the study was conducted in 2019. Is it possible to find smoking statistics of the relevant population in the more recent years? It applies to other paragraphs in the Introduction as well.

Methods

Page 8, line 13: are they never-smokers? If so, please rephrase the sentences here and in Table 1. The current sentence “Adult women who do not currently use any tobacco product” may imply that they used to be smokers.

Table 1: how were younger and older defined?

Results

Box 1: The sentences at the end were cut off.

Box 2: Statements 2.8 and 2.9 seem to be missing, as they were referenced in the main text Page 13, line 36.

Box 3: Same comment, the last few statements are missing even though they were referenced in the main text.

Box 4: 4.19-4.21 were missing.

**Reviewer**	**2**
**Name**	**ul Abidin, Muhammad**
**Affiliation**	**University of Sargodha, Sociology and Criminology**
**Date**	**19-Nov-2025**
**COI**	**None**

Review Report

Manuscript ID: bmjopen-2025-110684

Recommendation: Major Revisions

- This paper tackles a hugely relevant and timely public health concern: the increasing normalization of female smoking in an urban area of China. With a qualitative design and a very large sample (N = 288 in three large cities), the study stands well placed to provide insights into smoking behaviors, stigma and changing norms by gender. Ethical approval and the exhaled carbon monoxide monitoring to verify smoking status helped make the research more credible. In general, tobacco control policy and gender-specific prevention strategy efforts are highly impacted by this topic.
- The study strengths are in the investigation of an important but less well-known issue which is likely to impact women’s health. The large circle of participants lends itself to

different perspectives, especially with regard to smoking concealment, misinterpretation of health hazards, targeted marketing of tobacco to women, etc. The themes that we draw out will be meaningful and relevant to policymakers as well as scholars.

- The strengths of the paper are acknowledged but there are some methodological and reporting issues that need to be amended to meet the qualitative standards of BMJ Open. The manuscript does not articulate its analytical paradigm clearly enough.
- The study shows that a thematic analysis has been specified but gives little detail in regard to how the coding from the data led to the themes. The absence of a reflexivity statement noting the role and assumptions of the researcher and possible effect on the data skews the transparency of the analysis. To better alignment with SRQR qualitative reporting requirements, clearer justification of analytic decisions and improved integration of participant quotations is essential.
- Using WeChat as a recruitment strategy may create bias against younger high-tech urban people. The authors do concede that the findings may not be transferable to rural areas. However, they have not explored how that limits the overall interpretative reach of the study. The title and throughout the manuscript should reflect “urban China” to enhance accuracy and transparency.

- I have another concern is the inclusion of male focus groups. Because the research aims focus on women’s smoking experiences, the male perspectives seem somewhat peripheral, and occasionally overrepresented, in the Discussion. The authors should clarify if males’ data is supplementary or essential to their interpretation. Otherwise, male results will distract from core analytical interpretation.
- Some interpretive aspects such as those associated with Diffusion of Innovations Theory should be better anchored in focus group narrative content. Also, there are moments in the text that suggest causation (e.g., between increased marketing and increased acceptance) which need hedging or evidence.
- In China, the use of e-cigarettes and heated tobacco have grown rapidly, especially among young women but the discussion is mostly focused on cigarettes. We need clarification on whether and how these products made their way into the dataset and were subsequently analyzed.

- A table showing smoking by age and city would be useful for improving comprehension. Quotations from participants should be woven into the theme narrative, not in boxes.

Conclusion and Recommendation

This study may contribute to the literature on increasing tobacco use among females in more urbanized social contexts in China. But I think, **major revisions** are required to enhance the transparency of the methods, reinterpret the piece and improve the quality of reporting. The manuscript will be in good form for publication in BMJ Open after these changes.

VERSION 1 - AUTHOR RESPONSE

Comments from Reviewer: 1

Abstract

Comment 1: Please spell out any abbreviations when they first appeared, i.e. what does CO stand for in Page 1, line 12?

Response: Thank you for pointing this out. We have spelled out the abbreviation and replaced "CO" with "carbon monoxide" to ensure clarity.

Introduction

Comment 2: Page 6, line 16: The smoking rates or smoking attempt rate were reported in publications in the 2010s, and the study was conducted in 2019. Is it possible to find smoking statistics of the relevant population in the more recent years? It applies to other paragraphs in the Introduction as well.

Response: Thank you for the insightful comment. We have used two more recent data sources from 2019 to enhance the relevance of the cited evidence for the section you referred to and updated several references across the Introduction. Nevertheless, contemporary research specifically focusing on female smoking remains limited, particularly in recent years. Therefore, some earlier seminal publications were retained as they continue to provide essential context for understanding this under-researched population.

Some updated references are as follows:

20 Zhao Y, Di X, Li S, et al. Prevalence, frequency, intensity, and location of cigarette use among adolescents in China from 2013–14 to 2019: Findings from two repeated cross-sectional studies. Lancet Reg Health – West Pac. 2022;27. doi: 10.1016/j.lanwpc.2022.100549

21 Zhang M, Yang L, Wang L, et al. Trends in smoking prevalence in urban and rural China, 2007 to 2018: Findings from 5 consecutive nationally representative cross-sectional surveys. PLOS Med. 2022;19:e1004064. doi: 10.1371/journal.pmed.1004064

3 Allen AM, Oncken C, Hatsukami D. Women and Smoking: The Effect of Gender on the Epidemiology, Health Effects, and Cessation of Smoking. Curr Addict Rep. 2014;1:53–60. doi: 10.1007/s40429-013-0003-6

5 Abraham M, Alramadhan S, Iniguez C, et al. A systematic review of maternal smoking during pregnancy and fetal measurements with meta-analysis. PLOS ONE. 2017;12:e0170946. doi: 10.1371/journal.pone.0170946

Methods
Comment 3: Page 8, line 13: are they never-smokers? If so, please rephrase the sentences here and in Table 1. The current sentence "Adult women who do not currently use any tobacco product" may imply that they used to be smokers.
Response: Thank you for the suggestion. This group indeed refers to never-smokers who have never experimented with any tobacco products. We have revised the text and Table 1 accordingly to avoid potential ambiguity.

Comment 4: Table 1: how were younger and older defined?
Response: Thank you for raising this point. For generalizability of patterns, focus groupings were arranged by age (18-30, 30+) and other variables, we now explicitly indicate in the Methods section that:
Across the four interview categories, we purposively formed multiple groups, with half comprising participants aged 18–30 and the other half comprising participants aged over 30.
We have also removed the "younger" and "older" from Table 1 to avoid potential confusion.

Results
Comment 5: Box 1: The sentences at the end were cut off.
Comment 6: Box 2: Statements 2.8 and 2.9 seem to be missing, as they were referenced in the main text Page 13, line 36.
Comment 7: Box 3: Same comment, the last few statements are missing even though they were referenced in the main text.
Comment 8: Box 4: 4.19-4.21 were missing.
Response: We appreciate the reviewer for pointing this out. The missing content was due to an oversight in the previously uploaded file. To avoid such issues and better present quotations, we have removed the use of Boxes and integrated all quotations directly into the Results section. We have also carefully checked the revised manuscript to ensure that no quotations are missing.

<u>Comments from Reviewer: 2</u>

Comment 1: This paper tackles a hugely relevant and timely public health concern: the increasing normalization of female smoking in an urban area of China. With a qualitative design and a very large sample (N = 288 in three large cities), the study stands well placed to provide insights into smoking behaviors, stigma and changing norms by gender. Ethical approval and the exhaled carbon monoxide monitoring to verify smoking status helped make the research more credible. In general, tobacco control policy and gender-specific prevention strategy efforts are highly impacted by this topic.
Response: We sincerely thank the reviewer for the positive and encouraging comments.

Comment 2: The study strengths are in the investigation of an important but less well-known issue which is likely to impact women's health. The large circle of participants lends itself to different perspectives, especially with regard to smoking concealment, misinterpretation of health hazards, targeted marketing of tobacco to women, etc. The themes that we draw out will be meaningful and relevant to policymakers as well as scholars.
Response: We appreciate the recognition of the study's contribution and thank the reviewer for the comments.

Comment 3: The strengths of the paper are acknowledged but there are some methodological

and reporting issues that need to be amended to meet the qualitative standards of BMJ Open. The manuscript does not articulate its analytical paradigm clearly enough.
Response: Thank you for highlighting this important point. We have now revised the Methods section to more explicitly describe our analytical approach. The revised texts were presented in response to comment 4.

Comment 4: The study shows that a thematic analysis has been specified but gives little detail in regard to how the coding from the data led to the themes. The absence of a reflexivity statement noting the role and assumptions of the researcher and possible effect on the data skews the transparency of the analysis. To better alignment with SRQR qualitative reporting requirements, clearer justification of analytic decisions and improved integration of participant quotations is essential.
Response: Thank you for these valuable comments. We have substantially revised the Methods section to provide a clearer description of how coding was conducted and how themes were developed. A concise reflexivity statement has also been added. In addition, participant quotations have been fully integrated into the Results to enhance transparency and better align the manuscript with SRQR qualitative reporting standards.

The revised Methods section is as follows:
The focus group transcripts and accompanying field notes were analyzed using thematic analysis, as described by Braun and Clarke.[22] First, two researchers familiarized themselves with the data by reading all the transcripts line-by-line to generate initial thoughts on the data. A deductive coding framework was developed based on the focus group guides and applied to the transcripts. During coding, the analysts inductively generated additional descriptive codes to capture emergent topics. The coding framework was iteratively refined through team discussions, and overlapping or conceptually similar codes were revised, merged, or redefined as needed. Broader themes were then identified and defined by collating conceptually similar subcategories across the four participant groups and across all focus groups.
All transcripts were managed and coded in NVivo, and theme development was led by two analysts in consultation with the wider research team. The analysis team consisted of both male and female public health researchers trained in qualitative methods and tobacco control. To enhance reflexivity, the team discussed potential biases throughout the analytic process and grounded theme development closely in participants' accounts. Analyses were performed in the original Chinese; selected interview excerpts were translated into English for reporting. We followed the SRQR reporting guideline.[23]

Comment 5: Using WeChat as a recruitment strategy may create bias against younger high-tech urban people. The authors do concede that the findings may not be transferable to rural areas. However, they have not explored how that limits the overall interpretative reach of the study. The title and throughout the manuscript should reflect "urban China" to enhance accuracy and transparency.
Response: Thank you for this important comment. We wanted to clarify that while WeChat was one recruitment channel, we also recruited participants through offline community referrals to enhance diversity and reduce potential bias, which was missed in the original manuscript unintentionally. We agree that the findings are most applicable to urban contexts, and it was mentioned in the limitation of the original manuscript as follows:
This study was conducted in three major Chinese cities—Beijing, Changsha, and Shenzhen.

Therefore, the findings are primarily applicable to urban contexts and may not be generalizable to smaller cities or rural areas, where patterns of female smoking could differ.

Besides, in the revised manuscript, we have strengthened this point in the "Strengths and limitations of this study" section as follows:
As the study was conducted in urban settings, the findings may not be transferable to rural areas with different social norms and tobacco environments.

We have also checked the title and relevant parts of the text to explicitly reflect "urban China" for greater accuracy and transparency.

Comment 6: I have another concern is the inclusion of male focus groups. Because the research aims focus on women's smoking experiences, the male perspectives seem somewhat peripheral, and occasionally overrepresented, in the Discussion. The authors should clarify if males' data is supplementary or essential to their interpretation. Otherwise, male results will distract from core analytical interpretation.
Response: Thank you for raising this important point. Male focus groups were included to provide additional context on societal attitudes toward women's smoking, as we believed that understanding broader gender norms requires both women's lived experiences and men's perceptions. Their data were intended as supplementary rather than essential to the core analysis.
In the Results section, male perspectives were used sparingly—for instance, only one male quotation was included—and similar views were expressed within the women's groups. Relevant quotations are presented below:
But it seems that smoking among women, especially younger women, has increased. At least in my mother's generation, women didn't smoke. But now I do see some women in their 30s or 40s who smoke, and it even comes across as quite 'trendy'. [male, 30+, Changsha]
People are more open now. I think young people don't really care if women smoke. But the older generation, like my father's generation, still hold the view that it's acceptable for men to smoke but not for women. [female smoker, 30+, Beijing]

We now clarify that male data serve a contextual and supportive role, and we have reviewed the Discussion to ensure that women's experiences remain the primary analytic focus.

Comment 7: Some interpretive aspects such as those associated with Diffusion of Innovations Theory should be better anchored in focus group narrative content. Also, there are moments in the text that suggest causation (e.g., between increased marketing and increased acceptance) which need hedging or evidence.
Response: Thank you for this insightful comment. Upon careful consideration, we concluded that Diffusion of Innovations Theory may not be the most appropriate framework for interpreting our data, as we did not observe evidence of a clear diffusion pattern (e.g., urban-to-rural spread) or characteristics of "early adopters." Initiation appeared in both younger and older women, including those who started smoking after retirement. For this reason, we have removed interpretations that relied on Diffusion of Innovations Theory.

We also reviewed the parts of the manuscript where wording may have implied causation and have revised these statements to use more cautious, evidence-aligned language. The relationship between increased marketing and increased acceptance of female smoking was supported by relevant literature now.

The increased acceptance of female smoking may also be partly attributed to the growing tobacco marketing targeting women.[13] Advertising that associates smoking with women's liberation, independence, and equality with men has likely played a significant role in reshaping societal views.[11] Some female smokers in our study reported smoking "feminised cigarette products" and associated smoking with symbols of independence, which could be a direct result of such marketing campaigns. This suggests that social marketing of tobacco, which portrays smoking as a symbol of empowerment for women, may contribute to the normalization of female smoking by altering both descriptive norms (by increasing visibility of female smokers) and injunctive norms (by reducing social disapproval).[9]

References:

9 Hitchman SC, Fong GT. Gender empowerment and female-to-male smoking prevalence ratios. Bull World Health Organ. 2011;89:195-202. doi: 10.2471/BLT.10.079905
11 Amos A, Haglund M. From social taboo to "torch of freedom": the marketing of cigarettes to women. Tob Control. 2000;9:3–8. doi: 10.1136/tc.9.1.3
13 Tobacco Atlas. Tobacco Industry Targets Women and Girls as Next Generation of Smokers. Tob. Atlas. 2020. https://tobaccoatlas.org/tobacco-industry-targets-women-and-girls-as-next-generation-of-smokers/ (accessed 28 April 2025)

Comment 8: In China, the use of e-cigarettes and heated tobacco have grown rapidly, especially among young women but the discussion is mostly focused on cigarettes. We need clarification on whether and how these products made their way into the dataset and were subsequently analyzed.
Response: Thank you for highlighting this important issue. As noted in our limitations, our study did not collect sufficient information on other tobacco products, such as e-cigarettes or heated tobacco products, and therefore these products were not systematically captured or analyzed in our dataset. We agree that their rising use, particularly among young women, is highly relevant, and future research should examine these products to provide a more comprehensive understanding of women's tobacco use patterns in China.

Comment 9: A table showing smoking by age and city would be useful for improving comprehension. Quotations from participants should be woven into the theme narrative, not in boxes.
Response: Thank you for the helpful suggestion. We have added a new table showing the distribution of focus groups by city and participant type to improve contextual understanding (Table 2). Regarding age, half focus groups for each categories comprised participants aged 18–30 and the other half comprised participants aged over 30, which has been described in the revised Method section.
Table 2. Distribution of focus groups by city and participant composition

City	Women (smokers)	Women (never-smokers)	Women (ex-smokers)	Men	Total
Beijing	4	2	2	2	10
Changsha	4	2	2	2	10
Shenzhen	4	2	2	0	8
Total	12	6	6	4	28

In response to the second point, we have removed all Boxes and woven participant quotations directly into the thematic narrative within the Results section to enhance clarity and alignment with qualitative reporting standards.

Other significant updates:

We updated the funding statement as one of the co-authors (XY) is affiliated with the funder, and the funder facilitated the involvement of a qualitative research expert (JZ, not listed as author) who contributed data collection.

However, we declare the funder had no role in data coding, data analysis, interpretation of findings, manuscript preparation, or the decision to submit the article for publication. All analyses, interpretations, and conclusions are solely the responsibility of the authors, LX had final responsibility for the decision to submit for publication.
